# Standardized Low-Power Wireless Communication Technologies for Distributed Sensing Applications

**DOI:** 10.3390/s140202663

**Published:** 2014-02-10

**Authors:** Xavier Vilajosana, Pere Tuset-Peiro, Francisco Vazquez-Gallego, Jesus Alonso-Zarate, Luis Alonso

**Affiliations:** 1 Internet Interdisciplinary Institute (IN3), Universitat Oberta de Catalunya (UOC) C/Roc Boronat 117, Barcelona 08018, Spain; E-Mail: peretuset@uoc.edu; 2 Berkeley Sensors and Actuators Center, University of California Berkeley 403 Cory Hall #1774, Berkeley, CA 94720, USA; 3 M2M Department, Centre Tecnologic de Telecomunicacions de Catalunya (CTTC) Av. Carl Friedrich Gauss 7, Castelldefels 08860, Spain; E-Mails: francisco.vazquez@cttc.es (F.V.-G.); jesus.alonso@cttc.es (J.A.-Z.); 4 Signal Theory and Communications Group, Universitat Politecnica de Catalunya (UPC) Av. Esteve Terradas 7, C4-204, Castelldefels 08860, Spain; E-Mail: luisg@tsc.upc.edu

**Keywords:** low-power wireless, wireless sensor networks, radio frequency identification, medium access control, time-slotted channel hopping, DASH7 alliance mode

## Abstract

Recent standardization efforts on low-power wireless communication technologies, including time-slotted channel hopping (TSCH) and DASH7 Alliance Mode (D7AM), are starting to change industrial sensing applications, enabling networks to scale up to thousands of nodes whilst achieving high reliability. Past technologies, such as ZigBee, rooted in IEEE 802.15.4, and ISO 18000-7, rooted in frame-slotted ALOHA (FSA), are based on contention medium access control (MAC) layers and have very poor performance in dense networks, thus preventing the Internet of Things (IoT) paradigm from really taking off. Industrial sensing applications, such as those being deployed in oil refineries, have stringent requirements on data reliability and are being built using new standards. Despite the benefits of these new technologies, industrial shifts are not happening due to the enormous technology development and adoption costs and the fact that new standards are not well-known and completely understood. In this article, we provide a deep analysis of TSCH and D7AM, outlining operational and implementation details with the aim of facilitating the adoption of these technologies to sensor application developers.

## 1. Introduction

A major challenge to implement distributed sensing applications lays in the selection of the appropriate wireless technology, as the communication requirements are not the same for each application [1-3]. In general, the collection of sensor data from nodes can be classified into two categories according to the periodicity of the samples being collected. First, are periodic sensing applications in which sensors are sampled continuously at a given rate. This is the case of control loops that require having continuous information about the status of each sensor to decide which action to take. Second, are on-demand sensing applications in which all sensor data need to be collected when required. This is the case of the scenarios that require obtaining information from all sensors at a given instant of time. In addition to the communication requirements, there are other aspects that need to be considered when selecting an appropriate wireless technology for a distributed sensing application. As nodes typically operate using batteries, this mandates the use of low-power wireless technologies to enable operation for several years. Low-power wireless technologies are achieved through strict duty cycling of the radio transceiver, as these are the elements that consume most of the node's energy. Such control is conducted at the medium access control (MAC) layer, as it is ultimately responsible for managing the operation of the transceiver.

Over the last few decade, standards for low-power wireless communications have been developed to cope with the requirements of different sensing applications [4]. For example, the IEEE (institute of electric and electronic engineers) 802.15.4 standard [5] was published in 2003 for WPAN (wireless personal area networks), whereas the ISO (international organization for standardization) 18000-7 standard [6] was developed around 2004 for active RFID (radio frequency identification). Both protocols define the lowest layers of the communications protocol stack, *i.e.*, the physical and data-link layers, and depend on other protocols, e.g., ZigBee or RPL (IPv6 routing protocol for low-power and lossy networks) [7] and CoAP (constrained application protocol) [8], to provide a complete stack. IEEE 802.15.4 is more suitable for periodic data collection scenarios, where a node monitors the physical quantities at a given rate and reports the data periodically to a coordinator. This is due to the fact that, despite that the GTS (guaranteed time slot) can be adapted dynamically, changing the number of dedicated slots is challenging, because traffic bursts cannot be predicted. Contrarily, ISO 18000-7 is more suited for on-demand data collection scenarios, where the sensors need to be collected at once. These protocols have demonstrated the applicability of sensing distributed technologies, but suffer from different problems that limit their applicability, such as low network reliability, due to interferences, and high energy consumption under traffic load, due to channel access inefficiency.

To overcome these limitations, new wireless communication technologies specifically targeted at low-power scenarios have been already or are in the process of being standardized. This is the case of TSCH (time-slotted channel hopping) in IEEE 802.15.4e [9] and D7AM (DASH7 alliance mode) [10] in the DASH7 Alliance. TSCH is a protocol targeted at periodic sensing applications, whereas D7AM is more suitable for on-demand sensing applications. However, despite the current efforts to develop and standardize such low-power wireless technologies, few vendors are already implementing and selling these latest standards. In fact, most vendors are still sticking to earlier wireless technologies, such as ZigBee, due to the the inertia of introducing new protocols to slowly adopting markets, low volumes for hardware manufacturers and the costs of implementing quickly evolving protocols. Moreover, most open source projects that are being developed today are also based on earlier wireless technologies that do not meet the low-power requirements of today's sensing applications.

Taking that into account, in this article, we explore the implementation of state-of-the-art low-power wireless communication technologies for sensing applications and outline the key challenges and difficulties that need to be addressed with the aim of boosting and facilitating the development of these wireless technologies. Specifically, we contribute to this objective in three different ways: first, by introducing the operation of TSCH and D7AM in a way that is easy to understand and that targets the application requirements; second, by presenting the hardware and software requirements to implement both TSCH and D7AM; and third, by identifying the scope and suitability of TSCH and D7AM according to the sensing requirements.

The remainder of the article is organized as follows. Section 2 summarizes the operation of both TSCH and D7AM. Section 3 discusses the requirements to implement TSCH using commercial hardware. Section 4 discusses the requirements to implement D7AM using commercial hardware. Section 5 discusses the suitability of TSCH and D7AM according to different sensing application profiles. Finally, Section 6 concludes the article.

## 2. Low-Power Wireless Technologies

This section presents the emerging technologies for low-power wireless communications. First, we present time-slotted channel hopping (TSCH). Second, we present DASH7 Alliance Mode (D7AM).

### 2.1. Time-Slotted Channel Hopping

Time-slotted channel hopping (TSCH) is a MAC protocol aimed at periodic sensing scenarios and the core of numerous low-power wireless standards for industrial applications, such as ISA (international society of automation) 100.11a [11], WirelessHART [12] and IEEE 802.15.4e [9]. As the most recent standard, IEEE 802.15.4e was published in 2012 as an amendment to the medium access control (MAC) protocol defined by the IEEE 802.15.4-2011 [13] standard.

The IEEE 802.15.4 physical (PHY) layer is defined to operate in three different ISM (industrial, scientific and medical) bands. The 868.0-868.6 MHz band, which defines three communication channels, is only available in Europe, whereas the 902-928 MHz band, which can be divided into up to thirty channels, is only available in North America. Finally, the 2,400-2,483.5 MHz band is a true worldwide unlicensed band and offers sixteen channels. The IEEE 802.15.4-2006 physical layer revision defines the maximum data rates of the 868/915 MHz and 2,450 MHz bands, setting them to rates up to 100 kbps and 250 kbps, respectively. In addition, it defines different physical layers differentiated by the modulation method used. Most of them make use of a direct sequence spread spectrum (DSSS) technique that uses either binary (BPSK) or offset quadrature phase shift keying (OQPSK), at 868/915 MHz, and only OQPSK at the 2,450 MHz band.

At the medium access control (MAC) layer, the TSCH mode was designed to allow IEEE 802.15.4 devices to support a wide range of industrial applications. At its core, the medium access technique uses time synchronization to achieve ultra low-power operation and channel hopping to ensure high reliability [14]. This is very different from the “legacy” IEEE 802.15.4 MAC protocol and is thus better described as a “redesign”. TSCH does not amend the physical layer, thus being able to operate on any IEEE 802.15.4-compliant radio.

All motes in a TSCH network are synchronized, and time is divided in time slots. A time slot is long enough for a MAC frame of maximum size to be sent between two motes and for the receiver to reply with an acknowledgment (ACK) frame indicating correct reception. The duration of a time slot is not defined by the standard. With IEEE 802.15.4-compliant transceivers operating at the 2.4 GHz frequency band, a maximum-length frame of 127 bytes takes about 4.6 ms (milliseconds) to transmit; a shorter ACK takes about 1 ms. With a 15 ms slot (a typical duration) [15], this leaves 9.5 ms for radio management, packet handling and security operations.

Time slots are grouped into one or more slot frames. A slot frame continuously repeats over time. TSCH does not impose a slot frame size. Depending on the application needs, this can range from tens to thousands of time slots. The shorter the slot frame, the more often a time slot is repeated, leading to more available bandwidth and higher throughput, but also higher energy consumption.

A TSCH schedule instructs each mote what to do in every time slot: either receive, transmit or sleep. The schedule indicates, for each scheduled transmit (TX) or receive (RX) time slot, a *channelOffset* and the address of the neighbor with which to communicate. Once a mote obtains its schedule, it executes it:

- For every TX time slot, the mote checks whether there is a packet in the outgoing queue that matches the neighbor address specified in the schedule information for that time slot. If there is not, the mote keeps its transceiver switched off during the time slot. If there is a packet for that neighbor, the mote transmits it and can request an ACK back. In such case, the node keeps listening for the ACK after transmitting.
- For every RX time slot, the mote listens to the channel for possible incoming packets. If nothing is received after some listening period, the transceiver is switched off. If a packet is received, it is either addressed to the mote or is a broadcast message, and if it passes security checks, the mote can send back an ACK.

How the schedule is constructed, updated and maintained and by which component in the network is out of the scope of the IEEE 802.15.4e standard. Instead, these functions are addressed by the IETF (internet engineering task force) 6TiSCH working group [16].

The IEEE 802.15.4e standard can be considered the latest generation of ultra-lower power and reliable mesh networking solutions for low-power lossy networks (LLNs). Commercial networking solutions are available today in which motes consume few micro-amps on average with end-to-end packet delivery ratios over 99.999% [17]. Today, the OpenWSN protocol stack, developed at the University of California Berkeley [18], is the only existing open-source implementation of this protocol. The IEEE 802.15.4e TSCH focuses on the MAC layer only enabling a clean layering fit under an IPv6-enabled protocol stack for LLNs, running 6L0WPAN (IPv6 over low power wireless personal area networks) [7], RPL [19] and CoAP [8,20]. The IETF 6TiSCH working group [21] is currently defining the latest component for an open standard-based protocol stack to put together IPv6 technologies with the operational technologies rooted to the TSCH technique and, especially, to IEEE 802.15.4e.

### 2.2. DASH7 Alliance Mode

DASH7 Alliance Mode (D7AM) [10] is a new standard for low-power wireless communications developed by the DASH7 Alliance. Contrary to other existing low-power wireless technologies, D7AM defines all the layers of the OSI (open source interconnect) model, from the physical layer to the application layer, and is targeted at asynchronous communications. The goal of D7AM is to be simple, elegant and reliable for handling bursty, light data and asynchronous and transient usage models. This approach is referred to as BLAST (bursty, light, asynchronous, stealth and transitive), and this means that it is tightly tuned for dealing with inherently mobile devices that need to upload small bits of information reliably thanks to its excellent range, low-power and robustness features. A complete overview of D7AM can be found in [22].

To maximize the range of one-hop communications [23], D7AM operates at the 433 MHz band. The 433 MHz band, which provides 1.74 MHz of bandwidth, is available (almost) worldwide as an ISM band, thus not requiring a license to operate. The D7AM physical layer is organized into fifteen 108 kHz basic channels, which are combined into four channel classes. The base class provides a single 432 kHz channel. The normal class provides eight 216 kHz non-overlapping channels. The hi-rate class provides four 432 kHz overlapping channels, and finally, the blink class provides a single 648 kHz channel. D7AM uses a (G)FSK (gaussian frequency shift keying) modulation scheme, one narrowband (55 kbps) for the base and normal channels and one wideband (200 kbps) for the hi-rate and blink channels. The benefit of using FSK is that the modulated signal has a constant envelope and, thus, is less sensitive to amplitude distortion caused by the hardware or the channel. In addition, all channels are encoded to enhance the robustness of one-hop communications. The standard defines two encoding mechanisms, PN9 and FEC (forward error correction). The PN9 encoding scheme, implemented using a LSFR (linear feedback shift register), provides a statistically DC-free data stream, but introduces no processing gain. The FEC encoding scheme, implemented using a non-recursive convolutional code followed by a 4 × 4 interleaving matrix is optional and focuses on providing data integrity in environments with bursty errors caused by shadowing.

Regarding the data-link layer, D7AM supports both synchronous and asynchronous communication models. To support both communication models, the data-link layer is based on two well-known techniques: preamble sampling (PS) [24] and carrier sense multiple access (CSMA) [25]. In addition, all motes in the network share a common knowledge of time, the tick (ti), which is equal to 2^−10^ s or 0.976 ms and is the smallest amount of time at which events at the MAC layer can be resolved. However, there is no global network synchronization, as each clock may tick at a different rate, e.g., due to physical construction, aging or temperature drift.

Using PS, a node can trigger communications with another node or a group of nodes asynchronously. Nodes execute the *channel scan series*, which is an ordered list of time events at which nodes wake up and turn on the radio to receive a background frame. In order to trigger communications, the standard defines the *beacon transmit series*. The beacon transmit series is an ordered list of time events at which the node is expected to wake up and turn on the radio to transmit a background frame. Background frames include information regarding the time that the node is expected to wake up and the channel that it has to listen to. Both the *channel scan series* and the *background scan series* can be configured depending on the application requirements, e.g., to minimize latency or maximize battery duration.

Upon synchronization, nodes use foreground frames to exchange data using the two channel types available: non-guarded channels (NGC) and guarded channels (GC). NGCs use a pure ALOHA access mechanism, which does not require prior negotiation before transmission. Contrarily, GCs are based on non-persistent CSMA/CA (carrier sense multiple access/collision avoidance), which requires to check the status of the channel prior to transmission. If the channel is idle, based on assessing the energy present in the medium, the node waits for a certain amount of time and tries again. If the medium is still free, then the node starts transmitting the packet immediately. The standard defines three back-off mechanisms when the medium is busy: AIND (adaptive increase no division), RAIND (random adaptive increase no division) and RIGD (random increase geometric division). However, only RIGD, where transmission starts after a random interval and retransmission follows a l/(2*^n^*^+1^) decay function, is used in practice.

The physical layer and the data-link layer of D7AM are well known in the literature and, therefore, do not represent any major improvement over other existing low-power wireless technologies. However, the main difference between D7AM and other existing technologies is the query system implemented at the upper layers of the stack, which is highly integrated with the lowest layers. The query system enables to restrain the response of nodes to a query based on certain upper layer parameters. For example, during synchronization, the initiating node may indicate that the query is only for nodes that have a temperature sensor. Therefore, all the nodes that do not have a temperature sensor will not synchronize and attempt to reply to the subsequent queries. Once the nodes that have a temperature sensor are synchronized, the initiating node can request data only from nodes that have a certain temperature value, for example, below 25 °C. On subsequent requests, the initiating node can request data from the remaining nodes, indicating the appropriate temperature values, or collect the remaining nodes using a wildcard.

Thus, a D7AM network can be seen as a distributed database system for various sensor parameters, with certain nodes actuating as the database querier and the remaining nodes actuating as dynamic entries in the distributed database. Such a distributed database system can either be queried on demand, e.g., when triggered by an external agent, or configured to react to certain events, e.g., a temperature sensor going above a certain threshold.

## 3. Time-Slotted Channel Hopping

The aim of this section is to describe the major steps and challenges when implementing TSCH. The time synchronized nature of the protocol and its adaptation to commercial off-the-shelf transceivers impose some restrictions and considerations that are outlined in the following subsections.

### 3.1. Component Structure and Operation

**Figure 1.**
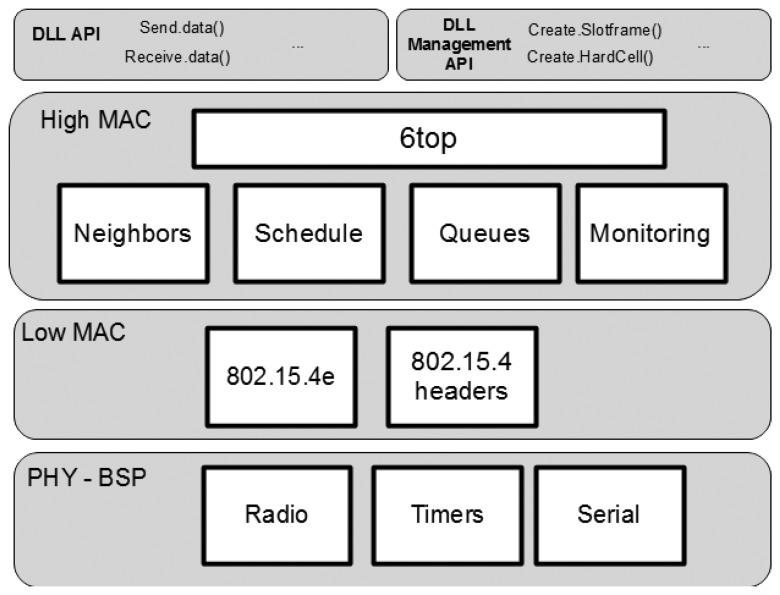
Component-based medium access control (MAC) organization.

The IEEE 802.15.4e TSCH does not define a component structure and management information base (MIB) organization, but only the overall MAC layer behavior, command-based API (application programming interface), message formats and the relevant personal area network information base (PIB) elements that are used by the MAC. The IETF 6TiSCH working group is defining the management interface and over-the-air messages to operate and configure the schedule of the TSCH MAC layer. Vilajosana *et al.* [15] define the minimal configuration of such a MAC layer to operate under a static schedule and the best effort routes guided by the routing protocol for low-power lossy networks (RPL) [19]. The component structure is open to implementers. In order to leverage the introduction of the technology, in this article, a component structure is defined. Figure 1 depicts a suitable component structure for the IEEE 802.15.4e implementation. The low MAC components are formed by a IEEE 802.15.4e module that is in charge of executing the state machine that guides the activities carried in a time slot, and the IEEE 802.15.4 component is used to manage headers and decouple time slot activities from header parsing. At the upper MAC layer, a 6 top component is in charge of managing the schedule, the neighbor table, the queues and the monitoring functions of the MAC layer.

Time slot timing is not defined in IEEE 802.15.4e. This is left open to implementers and can be configured dynamically by means of information elements (IEs) carried on enhanced beacon (EB) packets that are used to bootstrap the network. Determining those parameters is of utmost importance, as timing is critical in TSCH and the tuning of the MAC layers timing is time consuming. Table 1 presents a suitable timing for the low MAC layer as described by [15].

The presented component structure and timing is aligned with our previous work at the OpenWSN project [18], which constitutes the only existing open-source implementation of IEEE 802.15.4e available at the time of writing this article. The default MAC layer timing has been derived from experimenting with different platforms, including very old TelosB motes running at 4 MHz that required several milliseconds to handle serial port activity within a time slot. The separation of lower MAC from higher MAC is of utmost importance to decouple time slot intervals from higher layer traffic demand. In addition, the higher MAC layer is responsible for the management and frame control, while the lower MAC executes specific actions to send and/or receive packets, regardless of their type (data, control, *etc.*). The OpenWSN project supports a wide range of microcontrollers, including the Texas Instruments MSP430 family, the Freescale Kinetis family and the ST Microelectronics STM32F1 family, as well as radio transceivers, including the Texas Instruments CC2520 and the Atmel AT86RF23x. The latest addition is the Texas Instruments CC2538 SoC (system on chip), which includes an ARM Cortex M3 microcontroller and a radio transceiver compatible with the IEEE 802.15.4-2006 standard.

### 3.2. Implementation Details

**Figure 2.**
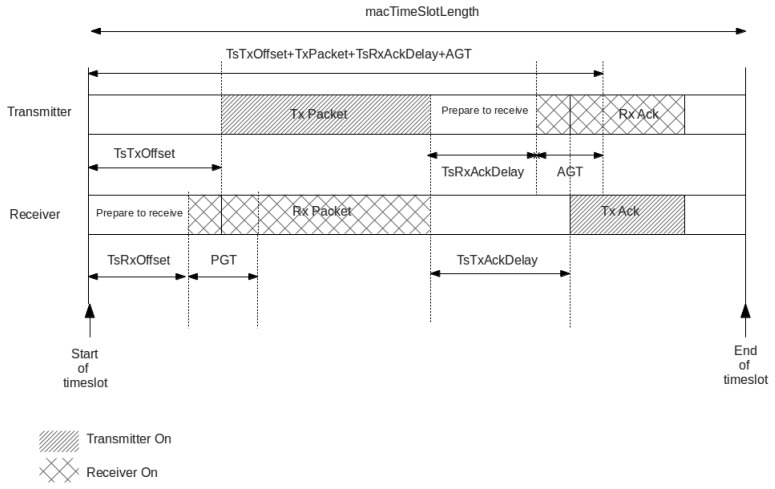
IEEE 802.15.4e time slot template. This defines the sequence of actions that take place in a time slot in both the sender and receiver nodes.

When implementing TSCH, several requirements need to be met by the hardware and the software components integrating the target platform. Mandatory requirements include the ability to control when a first bit of a packet leaves the radio, as well as to be able to detect when the first bit of an incoming packet reaches the transceiver. This requirement enables to timestamp incoming packets and determine the clock drift of the current node with respect to its parent, provided that transmission and reception occur at well-known time slots (see Figure 2). The software and hardware are closely related and specific timers with capture registers become very useful when connected to the pins of the radio, thus enabling automatic time stamping. As nodes must be aligned to a time source neighbor, source addresses need to be verified before synchronization to avoid alignment with other network siblings. The IEEE 802.15.4e TSCH suggests the use of different guard times to tolerate certain misalignment at the cost of a higher duty cycle. The IETF 6TiSCH working group recently defined some default values for guard times that enable to keep the network synchronized without a considerable synchronization overhead [15]. These values are typically close to a 1-ms guard time.

Manufacturing differences, operating temperature and voltage supply differences cause any clock source to tick at a slightly different frequency. For example, if a 32,768 Hz crystal is rated 30 ppm (parts-per-million), it drifts by at most 30 *us* (microseconds) every second with respect to a perfect clock. If two motes are equipped with this crystal, they can drift apart by as much as 60 *us* every second; one “going fast” and the other “going slow”. In this situation, it takes 16 s for those two motes to desynchronize by more than 1 ms. The IEEE 802.15.4e does not define the policy to maintain network synchronization *(i.e.*, how often and when synchronization packets are sent). Synchronization occurs when a child node communicates with its parent or it receives a packet from it. Enhanced beacons (EB) can be used for network bootstrapping at an initial synchronization step; however, the duration of their period has a certain impact on the energy consumption of the network. The problem comes when the network is idle and still and, due to clock drifts, it needs to re-synchronize every few tens of seconds. A trade-off exists between the use of dedicated keep-alive packets with an empty payload sent at a short period between nodes or the use of EBs. As defined by [15], a minimal TSCH network EB will carry several information elements (IE), and the packet length will be close to 60 bytes. In that case, it becomes more efficient to send keep alive (KA) packets periodically and to increase the EB period to reduce the energy consumption.

**Figure 3.**
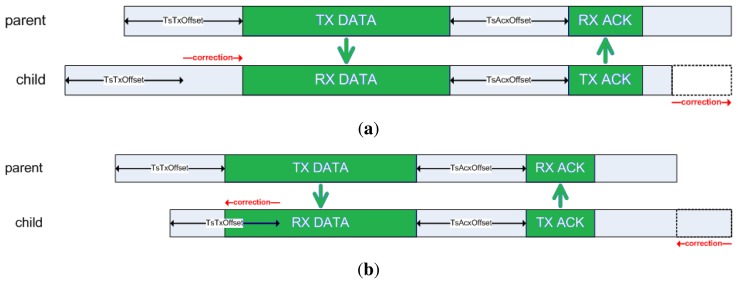
Packet synchronization diagram: (**a**) Packet synchronization: enlarging the time slot. (**b**) Packet synchronization: shortening the time slot.

**Figure 4.**
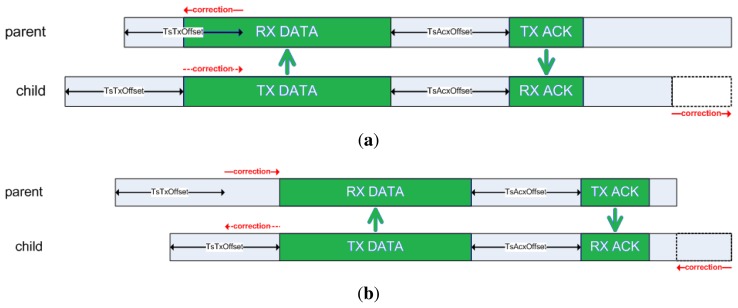
Acknowledge (ACK) synchronization diagram. (**a**) ACK synchronization: enlarging the time slot. (**b**) ACK synchronization: shortening the time slot.

As defined by IEEE 802.15.4e, the synchronization process is achieved by receiving a packet from a time source neighbor (referred to as a packet synchronization) or transmitting a packet to it (referred to as an acknowledgment synchronization). In the first case, a node (parent) will transmit a packet at exactly *TsTxOffset* from the beginning of the time slot (see Figure 2) to its child. When the first bit of the packet reaches the child's radio, it triggers a capture register on the child's timer, accounting for the relative time within the slot when the packet has been received. In an ideal world, the child node should capture exactly *TsTxOffset*. However, due to the clock drift, it will capture some slight difference value that indicates the current shift of this node with respect to its parent. This is depicted in Figure 3. To compensate for this lack of synchronization, the child node will change the length of its current time slot, so that it ends earlier or later, according to the shift detected. In the second case, a child node transmits a packet to its time source neighbor, and the parent node timestamps the time of arrival of the packet, detecting its offset with respect to the ideal time *TsTxOffset*. The parent then embeds a synchronization information element (SIE) in the ACK packet, including the time correction to be applied at the child node (see Figure 4). Upon reception of the ACK, the child node applies the time correction by changing the length of the current time slot.

**Figure 5.**
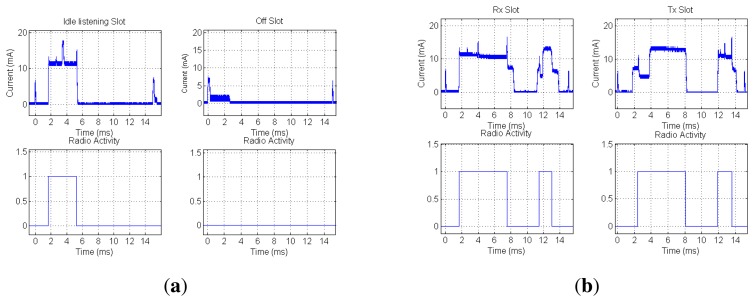
Measured current draw on a TelosB mote. (**a**) Idle listen and off slots; (**b**) transmission and reception slots.

A key consideration is to ensure that the TSCH MAC layer minimizes the radio duty cycle and also the overall platform energy consumption. Figure 5a shows the measured energy consumption of a mote during an inactive and idle listen slot. In turn, Figure 5b shows the measured energy consumed by a mote during a active slot, either transmitting or receiving a packet of 127 bytes in length. In both experiments, the current flow has been obtained by measuring the voltage drop across a 1-Ω shunt series resistor with an oscilloscope. It is important to note the importance of using the adequate low-power modes of the radio and microcontroller when there is nothing to be done during the slot or between actions in a slot. Key considerations when selecting a microcontroller are its ability to maintain a precise timer running in the lowest energy model that it is able to wake up the microcontroller and a ramp up time under 1 ms of the microcontroller when transitioning from deep sleep mode to normal run mode operation. To avoid timer impreciseness, a stable 32,768 Hz crystal should be used as the time source for the low-power timer peripheral. These considerations are fundamental to be able to implement TSCH MAC layers in off-the-shelf microcontrollers. As can be seen in Figure 5, inactive periods show an energy consumption close to 0 mA, as the radio and microcontroller are in deep sleep mode.

When designing a TSCH MAC layer, it is also important to decouple the actions that occur in a slot from the slot frame structure and management of the MAC layer. A higher MAC component is in charge of handling IPHC and higher layer packets and inserting them into a queue. In addition, it manages higher MAC services, such as neighbor discovery, scheduling and network bootstrapping by means of periodically sending EBs. In its turn, the lower MAC executes the activities that conform a slot, the beginning of which determines the type of slot (TX, RX, *etc.*) and reads from the queue to determine the action to be taken (e.g., checks if there is something to be transmitted in the case of a TX slot). This decoupling makes the lower MAC layer operate independently of the higher MAC, thus facilitating its implementation.

## 4. DASH7 Alliance Mode

The aim of this section is to describe the major steps and challenges to be faced when implementing D7AM in real hardware. The *ad hoc* nature of the protocol and its adaptation to commercial off-the-shelf transceivers impose some restrictions and considerations that are outlined in the following subsections.

### 4.1. Component Structure and Operation

**Figure 6.**
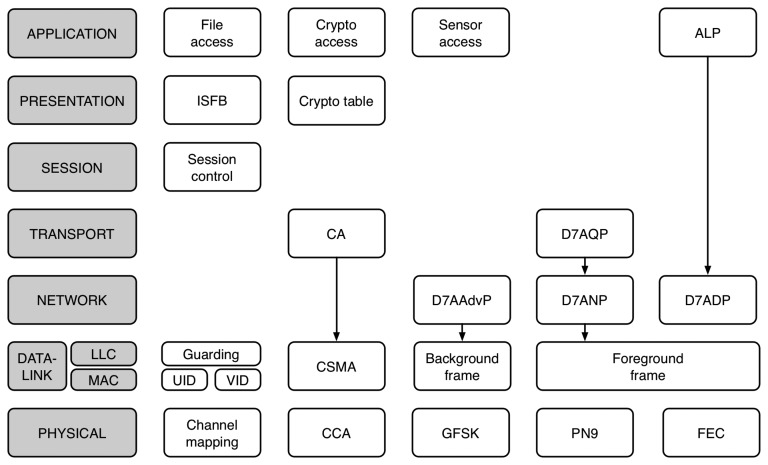
Stack organization of DASH7 Alliance Mode.

The D7AM is structured following the seven layers of the OSI model, as depicted in Figure 6. However, contrary to other protocols, where layer responsibilities are well-defined and isolated to ensure replaceability, the functionalities of each layer in D7AM are tightly coupled together to provide the means of configuring the protocol operation to suit application requirements. In that sense, the parameters defined in the indexed short file block (ISFB) at the application layer are used to configure the channel scan series and the beacon transmit series at the data-link layer, as well as the channel identifier at the physical layer. Moreover, the values of the ISFB can be remotely read or written, thus providing the means of reconfiguring the operation of a node. This enables to update the operation of the node on-demand to adapt to the specific requirements of each sensing application. For example, nodes of a distributed sensing application to monitor fire in a forest may be configured to operate using a push model, where they remain completely silent to save energy, unless a sensor event is detected. However, upon detecting fire, the nodes in the network can be reconfigured to provide periodic updates to enable the monitoring of the advance of the fire. Such an update in the network operation is only a matter of writing to the appropriate file in the ISFB, which can be done through standardized commands.

Despite the fact that layers are tightly coupled together, the D7AM standard does not define how each component of the stack is implemented. Typically, D7AM is implemented as a scheduler that executes tasks based on the current time and their relative priority. For example, a channel scan series at the data-link layer is represented as a series of tasks that are executed by the scheduler in order, one after the other. In addition to the channel identifier and the scan type, either background or foreground, each task in a channel scan series contains the scan timeout and the time until the next scan event. Using such data, the scheduler can determine which task to execute until a frame is successfully received or the process is stopped by an upper layer. Therefore, when a task begins execution, it configures the physical layer with the appropriate parameters, e.g., to receive a background or foreground frame in a given channel, and when it finishes, it triggers the appropriate follow-up actions, e.g., pass the packet to upper layers for further processing if a frame has been successfully received or execute the next scan event if no packet has been successfully received.

Currently, a reference implementation of D7AM supported by the DASH7 Alliance already exists. OSS-7 (open source stack) [22], lead by the CoSys-Lab (constrained systems laboratory) of the University of Antwerp, is an open source project that implements the D7AM standard and is licensed under an LGLP (GNU Lesser General Public License) license. The project is divided into each of the OSI layers, from the physical to the application layer, and two additional layers provide an abstraction of the hardware. The hardware abstraction layer (HAL) provides an abstraction of the microcontroller functionalities, whereas the radio abstraction layer (RAL) provides an abstraction of the radio transceiver capabilities. Regarding the HAL, OSS-7 currently supports the Texas Instruments MSP430 and the ST Microelectronics STM32L1 microcontroller. Regarding the RAL, OSS-7 currently supports the Texas Instruments CC1101 and the ST Microelectronics Spirit1 radio transceivers.

## 4.2. Implementation Details

One of the key aspects of D7AM is to implement certain aspects of the protocol using low-power microcontrollers, because the standard has been designed to be transceiver agnostic. Some transceivers already implement hardware that is compatible with features defined in the standard and, in such cases, the implementation is both time and energy efficient. Examples of such transceivers are the CC1101 of Texas Instruments and the Spiritl by ST Microelectronics. However, other transceivers do no implement such features, and thus, these need to be implemented in the microcontroller. One example is the CC430 system on chip (SoC) by Texas Instruments, which includes a 16-bit MSP430 microcontroller and a reduced version of the CC1101 transceiver.

**Figure 7.**
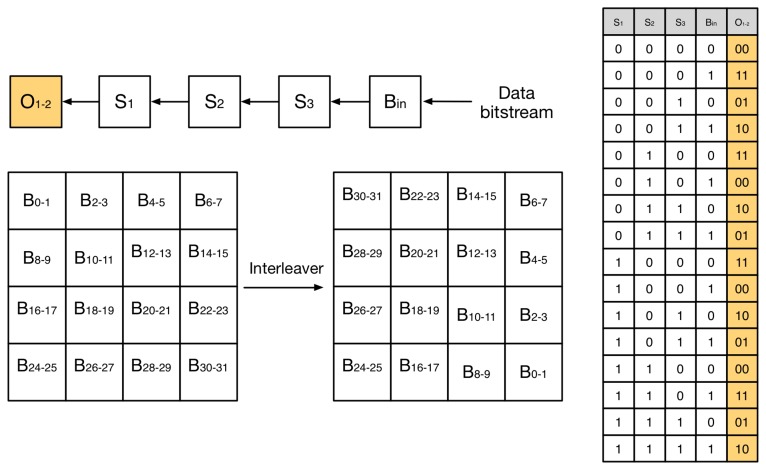
Forward error correction (FEC) coding schemes with the convolutional encoder and matrix interleaver.

One particular case is implementing the FEC encoding scheme at the physical layer, which is based on two subsystems connected in series, as depicted in Figure 7. First, a non-recursive convolutional encoder with a rate *r* = 1/2 and a constraint length *k* = 4. Second, a 4 × 4 matrix interleaver that separates adjacent data to ensure that bursty data errors caused by fading can be treated by the convolutional code error corrector. Compared to an uncoded channel, the FEC encoding scheme provides an asymptotic coding gain of 4.8 dB in an AWGN (additive white gaussian noise) channel [26], thus enabling to extend the communication distance under ideal conditions. However, such a gain is reduced in real-life conditions to 2-3 dB, due to channel impairments, such as fading and shadowing. The FEC encoding and decoding process is asymmetric, that is, the code to implement the coder and the decoder are different. During the encoding process, the microcontroller first shifts the data stream through a nibble mapper that selects the appropriate output given the input bits. Such a process can be implemented using a mapping table and, thus, is computationally efficient. Next, the output of the encoder is passed through the interleaver. The process is also computationally efficient, because the matrix transformation is small, and thus, it takes a few steps. Upon reception, the deinterleave process is similar to the interleave process. However, the decoding process is much more computationally intensive. Because the errors introduced by the channel are random, a Viterbi algorithm needs to be executed to select the path in the output stream that has the maximum likelihood decoding.

Considering the implementations of D7AM that are currently available [22], we have observed that PN9 encoding and decoding (as presented earlier) and the FEC encoding process can be implemented in software without any impact on system performance using an MSP430 microcontroller. However, the implementation of FEC decoding executing the Virterbi algorithm has an impact on the response time and energy consumption of the system. Other microcontrollers with more advanced computational characteristics, such as those based on the Cortex-M3 architecture, may be able to execute the Viterbi algorithm in real time. Thus, it is important to take into account the appropriate selection of the microcontroller and the radio transceiver when designing a low-power wireless system based on D7AM.

Another key aspect of implementing D7AM is the query system. In contrast to other low-power wireless technologies, where querying is typically done at the data-link layer based on the node address, D7AM enables to treat the network as a distributed database. Such an approach enables to query nodes in the network based on certain parameters at the application layer in a standardized way, e.g., trigger a response only from nodes that have a temperature sensor whose current value is, for example, between 0 °C and 24 °C. The benefits of such an approach are two-fold. First, only nodes that match the request will reply to the query, thus enabling to save the energy of the remaining nodes that are not involved in the query. Second, as only a certain group of nodes will engage in replying to a certain query and queries can be done incrementally, the congestion probability in the channel access mechanism is minimized. Thus, using the query system not only improves the significance of the received data, but also enhances the network performance and reduces the energy consumption of the nodes.

To implement the query system, a query template is defined at the transport layer. The query template is a variable length payload that is transported on top of the D7A network protocol (D7ANP) using foreground frames. Queries can be global or local depending on the nodes being addressed at the network layer, e.g., if the destination address at the data-link layer is broadcast or multicast, respectively. The variable length payload of a query template is composed of four fields: the compare length (one byte), the compare code (one byte), the compare mask (n bytes) and the compare value (n bytes). First, the compare length determines the length of the mask and the compare values fields in the query. Second, the compare code enables to select the compare type and operation. The supported compare types are non-null check (if the data exists), arithmetic comparison, string comparison and custom. In turn, the supported operations for arithmetic comparison are inequality, equality, less than, less or equal than, greater than and greater or equal than. Regarding the string comparison, the process is done bit by bit and a threshold can be defined to trigger a match. Third, the compare mask is the bitmask that will be applied to the compare value to trigger a match with the local data. Finally, the compare value is the data with which the local value will be compared after applying the compare mask.

Finally, another key aspect to implement D7AM consists in maintaining synchronization among the nodes that form the network to ensure proper protocol operation, e.g., the receiver is in receive mode before the transmitter starts transmitting. The protocol operation is based on a common knowledge of time, the tick or ti, which is defined as 32 ticks at 32.768 Hz (0.9765 ms) and needs to be shared by all nodes in the network. However, every node derives the tick reference from its own clock source, and thus, divergences may exist, due to static effects and the dynamic effects of crystals. Regarding static effects, differences in crystal construction can make each individual clock tick at different rates. For example, two clocks rated at 10 ppm can drift up to 20 ppm with respect to each other. Regarding dynamic effects, aging and temperature also effect the rate at which two clocks drift with respect to each other. Regarding aging, as the crystal vibrates, the friction affects its stability and accuracy, thus leading to relative drift. Regarding temperature, a crystal placed in an area 20 °C colder than another crystal will lead to a relative drift of around —15 *μ*s per second. However, it is important to take into account that D7AM operation does not have the same stringent requirements as TSCH because time synchronization is *ad hoc*. That is, synchronization happens every time a network event is triggered and only needs to be maintained for the duration of such an event, which typically lasts for a time shorter than clock drifts relative to each other.

## 5. Discussion

The former sections have presented TSCH and D7AM as protocols for low-power wireless communications and have described the main challenges to be faced when implementing the protocols in real hardware. In this section, we discuss in detail the suitability and applicability of each technology according to the different requirements of sensing applications.

### 5.1. Traffic Pattern and Node Mobility

Two key parameters in sensing applications are the traffic pattern and node mobility. Some sensing applications have deterministic traffic requirements, whereas others have an event-based nature or are subject to mobility. For example, control loops require a certain amount of bandwidth and a bounded latency, *i.e.*, 1 pps (packet per second) with a maximum latency of 10 ms, to ensure the correct operation of the device under control. Furthermore, periodic sampling applications, such as utility metering, have similar traffic requirements. Contrarily, event detection applications require a certain group of nodes to react to certain events, e.g., a node triggers an alarm when its temperature sensor goes above a certain threshold, or require to collect data only from a given subset of nodes present in the network, e.g., those nodes that have a temperature over a certain value. In both cases, the traffic pattern is not predictable, because the event that triggers communications has an *ad hoc* nature. Furthermore, when nodes are subject to mobility, the access to the network cannot be deterministic either. Therefore, the use of a network resource cannot be anticipated, and hence, the network cannot be scheduled efficiently.

Due to the characteristics of TSCH, where actions occur at known times and the network is fully scheduled, sensing applications with deterministic traffic requirements can benefit from such an approach. However, TSCH is not well suited for applications where the traffic pattern is non-deterministic, because of the overhead required to re-schedule the network every time a node has new requirements. Furthermore, because the traffic pattern is deterministic, a certain overhead is incurred by nodes when the network resources are assigned, but not utilized. The latter can be leveraged by very efficient vendor-specific hardware devices, but cannot be assumed as a general rule. Contrarily, D7AM is suitable for sensing applications where traffic is highly asynchronous, because the network is only triggered under certain circumstances. However, such an approach leads to an increased unreliability, due to contention effects that also increase energy consumption. This is especially critical in highly dense networks, because contention effects are more severe.

### 5.2. Single-Hop vs. Multi-Hop Communications

Another key requirement in sensing application is network coverage. TSCH is designed to support mesh networking, which enables to cover large areas by means of multi-hops. Multi-hop mesh networks enable flexible topologies and provide redundancy against environment changes, which is crucial in certain communication environments. Nevertheless, the realization of multi-hop transmissions requires a dense network deployment to ensure path availability. Unfortunately, this is an unrealistic assumption in many real scenarios. In addition, the end-to-end bandwidth is reduced, because the channel is busy and the delay increased, due to processing overhead. However, the per-hop average energy consumption of a given node can be lower, because the transmit power at each hop can be reduced. However, this comes at the price of increasing the overall network energy consumption, as many nodes are involved in the process of forwarding a packet to its final destination. Furthermore, multi-hop communications require routing, which increases energy consumption, due to network management overhead, and becomes less scalable. In addition, selecting the appropriate routing metric is application dependent and introduces significant complexity to the network.

Contrarily, D7AM is mainly designed to support star topologies by means of using single-hop communications, though it also supports multi-hops through an optional feature at the network layer, where certain nodes can forward packets to extend the network coverage. In single-hop communications, the transceiver characteristics, e.g., transmit power and sensitivity, are crucial, because they determine the communication range. D7AM operates in the 433 MHz band with lower data rates and optional channel encoding, which enables to have longer range communications, due to its better propagation and sensitivity characteristics. However, operating in the 433 MHz band has some downsides, as well. First, narrow-band channels are more subject to the effects of interference, both internal and external, and multi-path propagation. Second, due to the limitations in the available bandwidth, the channels in the 433 MHz band are not orthogonal, meaning that certain channels interfere with other channels in the same band. Third, regulations in the 433 MHz band are not harmonized world-wide, and, thus each application needs to be tailored depending on the specific country regulations.

### 5.3. Network Reliability

Network reliability is a major concern in industrial applications, even more than latency and energy consumption, because wireless communications are replacing wired buses that instrument critical systems. In that sense, it has been demonstrated that TSCH networks can provide 99.999% reliability [17], but this imposes certain constraints in the network design. Network density is very important in multi-hop networks, as network bottlenecks become single points of failure that channel hopping cannot combat. Thus, to achieve high reliability, TSCH networks require some redundant paths to relay data. Despite this consideration, TSCH has been designed to be very robust against multi-path effects and external interferences thanks to the use of slow channel hopping, short packets, scheduled communications and network synchronization, which ensures a very low collision probability even between hidden terminals.

The approach to provide network reliability in D7AM is radically different from TSCH. Instead of relaying on multi-hop communications, which requires to have a dense network, D7AM bases its operation on single-hop communications. To ensure that single-hop communications are reliable, the standard uses a robust modulation scheme combined with channel coding, which allows to achieve high packet delivery ratios, even when nodes are at the sensitivity edge. In addition, D7AM can use acknowledgments and retransmission to ensure the delivery of certain packets. Finally, D7AM can also provide reliability against internal and external interferences through slow channel hopping; each communication instance can take place at a different channel. However, slow channel hopping does not provide any advantage regarding multi-path propagation, because the channels used in D7AM are narrowband [23].

## 6. Conclusions

This paper has presented TSCH and D7AM, the latest generation of low-power wireless technologies suited for distributed sensing applications. Taking into account its characteristics, TSCH is more suitable for periodic sensing applications, where nodes are static and a dense deployment can be ensured. Contrarily, D7AM is more suitable for *ad hoc* sensing applications, where nodes are either mobile or static and need to upload small bits of information. The paper has focused on providing a deep overview of the protocols and deriving the implementation details to facilitate its adoption for industrial implementers. The article initially claimed that industries are not still shifting to these new standards because they are not well understood and the costs of their adoption are still high, due to the lack of open-source initiatives and knowledge that can be transferred. However, this situation is in the process of changing, thanks to projects, such as OpenWSN and OSS-7, which provide a reference implementation of such standards, as well as extensive documentation.

## Figures and Tables

**Table 1.** Time slot timing. TSCH, time-slotted channel hopping.

IEEE 802.15.4e TSCH Parameter	Value
TsTxOffset	4,000 *μs*
TsLongGT	2,600 *μs*
TsTxAckDelay	4,606 *μs*
TsShortGT	1,000 *μs*
Time slot duration	15,000 *μs*
